# Dynamic Analysis and Experiment of 6-DOF Compliant Platform Based on Bridge-Type Amplifier

**DOI:** 10.3390/mi11111024

**Published:** 2020-11-23

**Authors:** Chao Lin, Shan Zheng, Mingdong Jiang

**Affiliations:** State Key Laboratory of Mechanical Transmission, Chongqing University, Chongqing 400030, China; s_zheng@cqu.edu.cn (S.Z.); 201907021036@cqu.edu.cn (M.J.)

**Keywords:** bridge-type amplifier, dynamic analysis, piezoelectric actuator, compliant platform

## Abstract

In this paper, we establish a dynamic model of a six-degrees-of-freedom (6-DOF) compliant positioning platform based on bridge-type amplifiers. Based on the elastic beam theory and energy relationship, we derived the bridge-type amplifier’s dynamic model using the Lagrange equation. Then, we established a dynamic model of the compliant platform based on the equivalent mass and equivalent stiffness of the bridge-type amplifier, and the analysis formula of the natural frequency was derived. Finally, the analytical models of natural frequencies of the bridge-type amplifier and the compliant platforms were verified using the finite element analysis (FEA) method. Through modal experiments, the damping ratio and natural frequency were identified. Step response experiments in the X/Y direction and Z direction were performed. The phenomenon that the experimental results appeared to match the theoretical calculations indicates that the dynamic model was accurate.

## 1. Introduction

The compliant positioning platform driven by a piezoelectric actuator (PZA) is widely used in aerospace technology, Microelectronics Mechanical Systems (MEMS), biomedical engineering, optical engineering, and other modern precision engineering fields [1,2], taking advantage of the advantages of a PZA, such as the ultra-high displacement resolution, high-frequency response, and large output force [3,4], as well as the characteristics of a flexible hinge, such as high precision, fast response, and compact structure [5,6].

However, since the stroke of the PZA is small, it is only 0.1% of its length [2], generally several tens of microns. An amplifier is generally required to amplify the PAZ’s stroke to obtain the positioning platform driven by the PZA. The displacement amplifiers based on flexible hinges have the advantage of being without friction and with no gap. They are widely designed to amplify the output displacement of PZA [7]. Currently, the main displacement amplifiers include lever amplifiers [8], bridge-type amplifiers [9], rhombic amplifiers [10], and Scott–Russell amplifiers [11].

In recent years, micro/nano-positioning compliant platforms have experienced different structures ranging from a single degree-of-freedom (DOF) to multiple DOF due to their complete and essential application requirements. For example, Qu et al. [12] presented a 1-DOF large-range flexure-based platform with a remote-center-of-motion characteristic, composed of an outer remote-center-of-motion (RCM) guiding mechanism inner output-stiffness enhanced lever amplifier. Jiang et al. [13] developed an XY 2-DOF compliant positioning platform with stiffness modeling and performance analysis of input and output decoupling characteristics. 

Kenton et al. [14] proposed a 3-DOF tandem motion nano-positioning platform for scanning probe microscopy, which is continuously curved with a vertical rigid double hinge to guide the movement of the sample platform to minimize parasitic motion and off-axis effects. A parallel 2-DOF flexible platform was developed [15] for XY nano-positioning based on a multi-stage amplification mechanism consisting of a bridge-type amplifier, rhombic amplifier, and Scott–Russell amplifier. For optical instrument alignment, a precision 3-DOF vertical positioning platform was developed [16], driven by three PZTs, displacement-amplified by a bridge-type amplifier, and guided by three rotationally symmetric hinges. 

To meet the application requirements of higher degrees of freedom, Kang et al. [7] proposed a 6-DOF compliant platform for precision optical calibration systems. The platform used a 3-PPRRR (a tripod parallel mechanism with two prismatic pairs) triangular parallel structure, and the three XY 2-DOF piezoelectric positioning structure drove the compliant joint to achieve six degrees of freedom. A 6-DOF precision positioning system was proposed [17] consisting of two 3-DOF parallel mechanisms. Each parallel mechanism was driven by three PZAs and guided by three symmetrical T-shaped hinges and three elliptical flexible hinges. 

Most micro/nano-positioning compliant platforms can only be accurately positioned in the plane. The majority of the platforms that can achieve spatial 6-DOF positioning have serious displacement coupling problems. The 6-DOF positioning platform studied in this paper demonstrated a better decoupling performance, especially since the Z-direction was uncoupled [18]. In our previous work, the static and kinematics were explored, and this paper will continue to discuss the dynamics. There are also many studies on the dynamics of flexible hinges and compliant platforms.

The Lagrangian method was used to establish the dynamic equation of the 5-DOF positioning platform [19] based on the spatial motion of the rigid body. Du et al. [20] derived the natural frequency of a compliant platform with 2-DOF by accurately calculating the hinge’s equivalent mass. Considering the interaction between inertia and stiffness as determined by the size of the structure, Hongzhe et al. [21] established a dynamic model of the double parallel four-bar mechanism. Xu et al. [22] established a dynamic model of the bridge-type amplifier based on the Euler–Bernoulli beam theory and Lagrange equations. Lagrange equations were used in the above literature; however, system damping was not considered in the model. Instead, Dao et al. [23] considered the influence of damping and proposed a compliant platform with embedded strain gauges and a viscoelastic damper. Secondly, only the natural frequency model was established, and the dynamic model of the working state was not analyzed. In the working state, the compliant platform typically had the steps of input and output. Ling et al. [24,25,26] deduced a dynamic stiffness matrix of the flexible beam based on D’alembert’s principle to analyze all the kine-matic, static and dynamic performances of compliant mechanisms. Yu et al. [27] analyzed the inverse dynamics of the 8R compliant mechanism based on Structural dynamics to model the compliant mechanism. Go et al. [28] proposed a concept of a 3-DOF rotary vibration-assisted micropolishing system (3D RVMS) and determined the dynamic and static performances based on compliance matrix method and FEA. In this paper, we established a complex dynamic model of a 6-DOF platform and considered the damping dissipation in the process of establishing a dynamic model with the Lagrange equation. The step response, including the step displacement *S_p_*, the system settling time *t_s_*, and the system maximum overshoot *M_p_*, was solved using the fourth-order Runge–Kutta. The correctness of the theory was verified by finite element analysis (FEA) and experiments.

## 2. Dynamic Modeling

### 2.1. Platform Description

**Figure 1:**
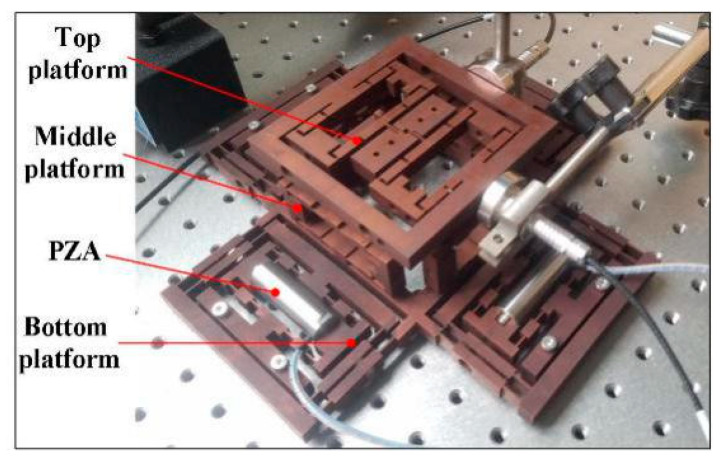
Structure diagram of the platform.

The 6-DOF micro/nano-positioning compliant platform of this study is based on bridge-type amplifiers, as shown in Figure 1, consisting of a top platform, a middle platform, and a bottom platform. The bottom platform is symmetrically distributed by the four bridge-type amplifiers in the X and Y directions and guided by four double parallelogram mechanisms to eliminate the displacement coupling in the X and Y directions. The middle four bridge-type amplifiers are used for Z-direction displacement positioning and rotational positioning around the X and Y axes. When four or two symmetric PZAs are simultaneously driven, the entire upper platform moves upward to achieve Z-direction displacement positioning. 

Conversely, when only one PZA is driven, the upper platform achieves deflection positioning around the X and Y axes. Two bridge-type amplifiers control the rotation of the platform around the *Z*-axis in the top platform. The proposed compliant platform was monolithically fabricated by Wire cut Electrical Discharge Machining (WEDM) technology, where the basic working principle was to continuously move fine metal wires as electrodes to perform pulse spark discharge to remove metal and cut the workpiece. In our previous work, the statics and kinematics of the platform were explored, and the dynamics continue to be discussed.

### 2.2. Dynamic Model of Bridge-Type Amplifier

**Figure 2:**
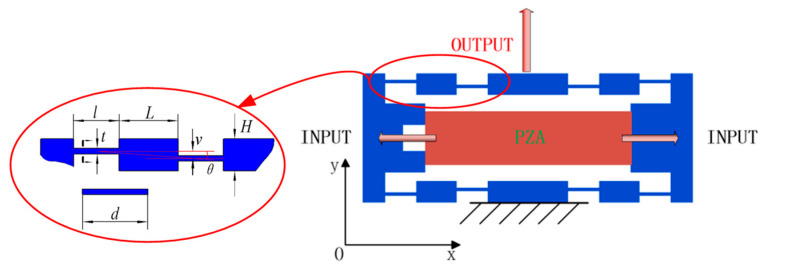
The principle and parameters of the bridge-type amplifier.

Compared with other types of amplification mechanisms, such as lever amplifiers, rhombic amplifiers, and Scott–Russell amplifiers, the bridge-type amplifier with a compact structure and large displacement amplification ratio is extensively adopted in engineering applications. The principle and relevant parameters of the bridge-type amplifier are shown in Figure 2. The input displacement and input force generated by the PZA in the X direction produced an amplified output displacement on the *Y*-axis. The hinge length is l, the thickness is t, the length of the connecting beam is L, the height is H, the angle between flexible hinges is θ, the spacing is v, and the mechanism’s width is d.

**Figure 3:**
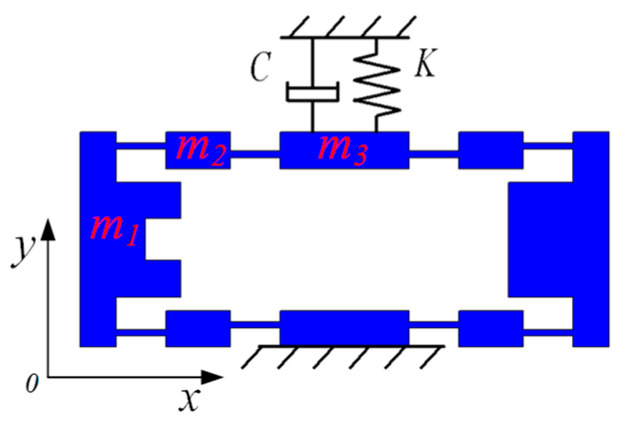
A dynamic model diagram of the bridge-type amplifier.

In a dynamic model based on the traditional PRBM (pseudo rigid body model) method, the damping of the compliant platform is typically ignored. However, when the vibration problem is studied, ignoring the hinge damping will not match the actual damping [19]. To reach an analytical model close to the actual value, a dynamic analytical model considering the influence of damping is explored in this paper. A schematic diagram of the dynamics model is shown in Figure 3.

In this paper, the Lagrange equation is used to model the dynamics of the bridge-type amplifier. Considering the action of damping force, this can be written as:

(1) $$\frac{d}{d_{t}}\left(\frac{\partial \left(T-V\right)}{\partial {\dot{q}}_{i}}\right)-\frac{\partial \left(T-V\right)}{\partial q_{i}}+\frac{\partial D}{\partial {\dot{q}}_{i}}=Q_{i}\left(j=1,2,\ldots ,k\right)$$

where *T* is the kinetic energy of the system; *V* is the potential energy of the system; *q_i_* is the generalized coordinates of the system; *D* is the damping dissipation function of the system, and *Q_i_* is the generalized force corresponding to the generalized coordinates.

As shown in Figure 3, the mass of the left and right rigid beams are *m*_1_, the mass of the four connecting beams is *m*_2_, and the mass of the intermediate output beam and the lower fixed beam is *m*_3_; therefore, the system kinetic energy can be calculated as:

(2) $$T=2\times \frac{1}{2}m_{1}{\left(\Delta \dot{x}\right)}^{2}+\frac{1}{2}m_{3}{\left(2\Delta \dot{y}\right)}^{2}+4\times \frac{1}{2}J{\left(\Delta \dot{\alpha }\right)}^{2}.$$

For the system potential energy *V*, the elastic potential energy is mainly considered. Since the deformation of the connecting beam is relatively small, only the tensile deformation and bending deformation of the flexible hinge are considered here, and the potential energy of the system can be calculated as:

(3) $$V=8\times \frac{1}{2}k_{1}{\left(\Delta l\right)}^{2}+8\times \frac{1}{2}k_{\theta 1}{\left(\Delta \alpha \right)}^{2}$$

where Δ*l* and Δ*α* are the tensile deformation amount and the bending deformation amount of the flexible hinge, respectively. *k*_1_, *k_θ_*_1_ are the tensile stiffness and bending stiffness of the flexible hinge, respectively, which can be written as:

(4) $$k_{1}=\frac{Edt}{l};k_{\theta 1}=\frac{Edt^{3}}{12l}.$$

When one output of the bridge amplifier is fixed, there is only one degree of freedom on the other output. Only one degree of freedom in the Y direction of the bridge amplifier is studied here, and the generalized coordinate *q* = 2Δ*y* can be taken. Then, Δ*x*, Δ*l* and Δ*α* can be shown as:

(5) $$\Delta x=\frac{q}{2A};\Delta l=\frac{q}{ck_{1}};\Delta \alpha =\frac{q}{4\left(L+l\right)}$$

where *A* is the amplification ratio, and *c* is the flexibility matrix unit, which were derived in our previous work [18].

(6) $$A=\frac{k_{1}k_{2}\tan\theta \left(2mk_{\theta 2}+nk_{\theta 1}\right)-12k_{\theta 1}k_{\theta 2}\tan\theta \left(2k_{2}+k_{1}\right)}{12k_{\theta 1}k_{\theta 2}\left(2k_{2}+k_{1}\right)+k_{1}k_{2}\tan^{2}\theta \left(2mk_{\theta 2}+nk_{\theta 1}\right)}$$

(7) $$c=\frac{m\sin\theta \cos\theta }{6k_{\theta 1}}+\frac{n\sin\theta \cos\theta }{12k_{\theta 2}}-\frac{2\sin\theta \cos\theta }{k_{1}}-\frac{\sin\theta \cos\theta }{k_{\theta 2}}$$

where *m* and *n* are the abbreviations of polynomials, which are expressed as:

(8) $$\left\{ \begin{array}{l} m=\frac{3{\left(L+l\right)}^{2}}{\cos^{2}\theta }-\frac{6(L+l)L}{\cos\theta }+4L^{2}\\ n=\frac{3{\left(L+l\right)}^{2}}{\cos^{2}\theta }-\frac{6(L+l)l}{\cos\theta }+4l^{2} \end{array}\right.$$

where *k*_2_ and *k_θ_*_2_ are the tensile stiffness and bending stiffness of the connecting beam, respectively, which can be expressed as:

(9) $$k_{2}=\frac{EdH}{L};k_{\theta 2}=\frac{EdH^{3}}{12L}$$

The input force *F* exists when the mechanism is in operation. The PZAs composed of stacked piezoelectric ceramics are very rigid and will not deform substantially during operation; thus, the output force can be considered constant. According to the principle of virtual work, the PZA driving force in the virtual displacement *δ_x_* can be shown as:

(10) $$\delta W=F\cdot \delta x=F\cdot \frac{\partial x}{\partial q}\cdot \delta q.$$

By utilizing Equations (1)–(10), the dynamic models of the bridge-type amplifier can be deduced as follows:

(11) $$\{\begin{array}{c} M_{e}\ddot{q}+C\dot{q}+K_{e}q=Q\\ M_{e}=\frac{m_{1}}{2A^{2}}+\frac{m_{2}\left(L^{2}+H^{2}\right)}{12{\left(L+l\right)}^{2}}+m_{3}\\ K_{e}=\frac{2}{{\left(L+l\right)}^{2}}\cdot \frac{K_{\theta 1}\cdot K_{\theta 2}}{K_{\theta 1}+K_{\theta 1}}\\ Q=\frac{F}{2A}\\ C=2\xi \sqrt{M_{e}K_{e}} \end{array}$$

where, *M_e_* and *K_e_* are the equivalent mass and equivalent stiffness of bridge-type amplifier, and *C* and *ξ* are the damping coefficient and damping ratio. Therefore, the natural frequency is expressed as:

(12) $$f_{n}=\frac{1}{2\pi }\sqrt{\frac{K_{e}}{M_{e}}}.$$

### 2.3. Dynamic Model of Platform

**Figure 4:**
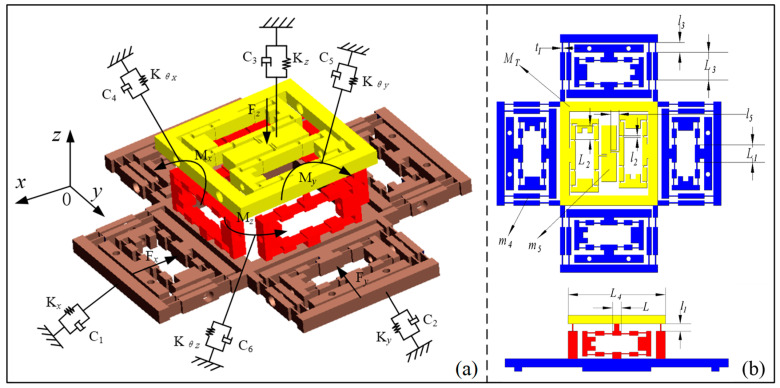
The parameters and dynamic model diagram of the platform. (**a**) The dynamic model diagram of the platform; (**b**) The parameters of the platform.

As shown in Figure 4, the entire structure of the compliant platform of this study was connected with a flexible hinge. In addition to the bridge-type amplifiers described in chapter 2.2, the bottom platform included the double four-bar guiding mechanism to prevent displacement coupling. Compared with the mechanism of the single-bridge approach [29], the proposed symmetrical and bidirectional structure effectively prevented large, coupled displacements. The platform dynamics model diagram is shown in Figure 4a, and the related parameters are shown in Figure 4b. The Lagrange equation was used to derive the dynamic model of the platform.

**Figure 5:**
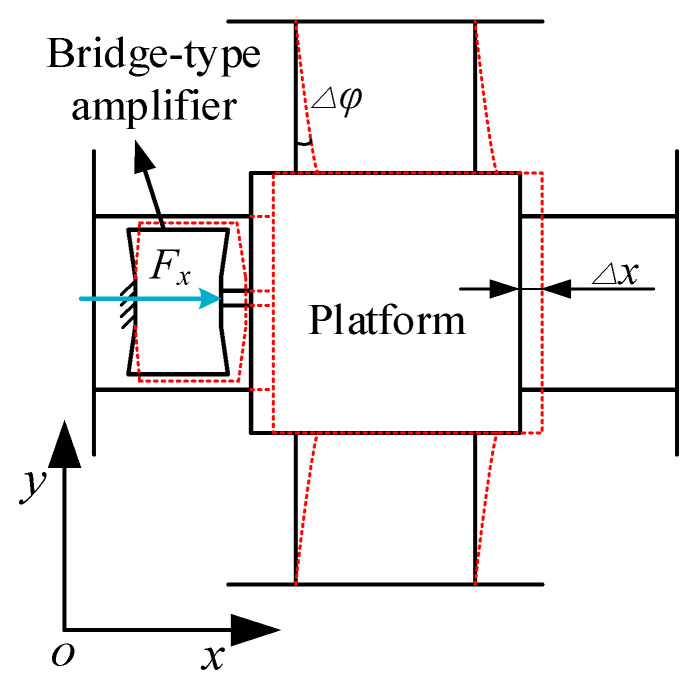
Schematic of translation in the X-direction.

As shown in Figure 5, when the platform has a displacement Δ*x* in the X-direction, the middle platform and the top platform have the same displacement Δ*x* due to no constraint. Due to the high lateral stiffness of the bridge-type amplifier, the displacement of the bridge-type amplifiers controlling the Y-shifting in the bottom platform is negligible indicating that only the rotation of the Y-direction double four-bar mechanism should be considered. From the geometric relationship, the angle of the double four-bar mechanism in the Y-direction can be expressed as:

(13) $$\Delta \phi \approx \tan\Delta \phi =\frac{\Delta x}{2l_{3}+L_{3}}$$

Therefore, the kinetic energy in the X direction can be expressed as:

(14) $$T_{x}=\frac{1}{2}\left(M_{M}+M_{T}+4M_{B}+8m_{4}\right)\Delta {\dot{x}}^{2}+M_{e}\Delta {\dot{x}}^{2}+4J_{4}{\left(\frac{\Delta \dot{x}}{2l_{3}+L_{3}}\right)}^{2}$$

where *M_M_*, *M_T_*, and *M_B_* are the mass of the middle platform, top platform, and the beam of the bottom platform, respectively; and *J_4_* is the moment of inertia of the connecting beam m_4_. 

When the platform has a movement in the X direction, the potential energy of the system mainly comes from the elastic potential energy of the two bridge-type amplifiers in the X direction, the tensile deformation of the two double parallelogram mechanisms in the X direction, and the bending deformation of the two double parallelogram mechanisms in the Y direction. This can be expressed as:

(15) $$V_{x}=K_{e}{\left(\Delta x\right)}^{2}+2k_{3}{\left(\Delta l_{3}\right)}^{2}+k_{g}{\left(\Delta x\right)}^{2}$$

where *k*_3_ and Δ*l*_3_ are the tensile stiffness and elongation of the hinge *l*_3_, which can be expressed as:

(16) $$k_{3}=\frac{Edt_{1}}{l_{3}};\;\Delta l_{3}\approx \frac{1}{2}\Delta x.$$

*K_g_* represents the bending stiffness of the double four-bar mechanism. According to reference [30], this can be computed as:

(17) $$k_{g}=\frac{12EI_{3}}{4l_{3}^{3}+6L_{3}l_{3}^{2}+3l_{3}L_{3}^{2}}.$$

Due to the symmetrical structure of the bottom platform, the kinetic energy and potential energy of the Y-direction translation are consistent with the X-direction.

**Figure 6:**
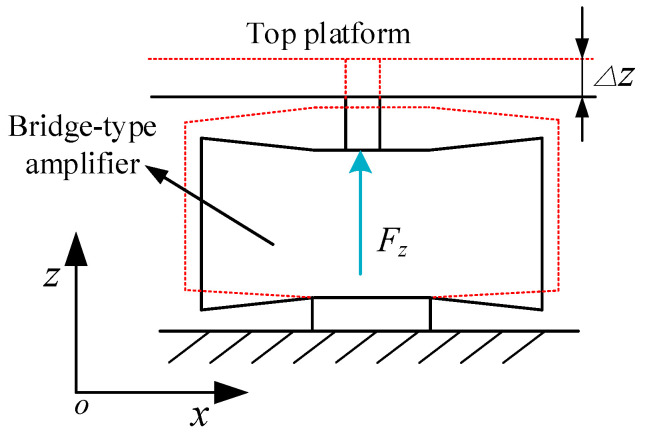
Schematic of translation in the Z direction.

As shown in Figure 6, when the platform is displaced by Δ*z* in the Z-direction, the middle four bridge-type amplifiers and the entire top platform are displaced. The kinetic energy of the system includes the kinetic energy of the entire upper platform and the kinetic energy of the four bridge amplifiers, which can be expressed as:

(18) $$T_{z}=\frac{1}{2}M_{T}\Delta {\dot{z}}^{2}+4\times \frac{1}{2}M_{e}\Delta {\dot{z}}^{2}$$

At the same time, the potential energy of the system is derived from the elastic potential energy of the four bridge-type amplifiers and the gravitational potential energy of the top platform, which can be denoted as:

(19) $$V_{z}=4\times \frac{1}{2}K_{e}\Delta z^{2}+M_{T}g\Delta z.$$

**Figure 7:**
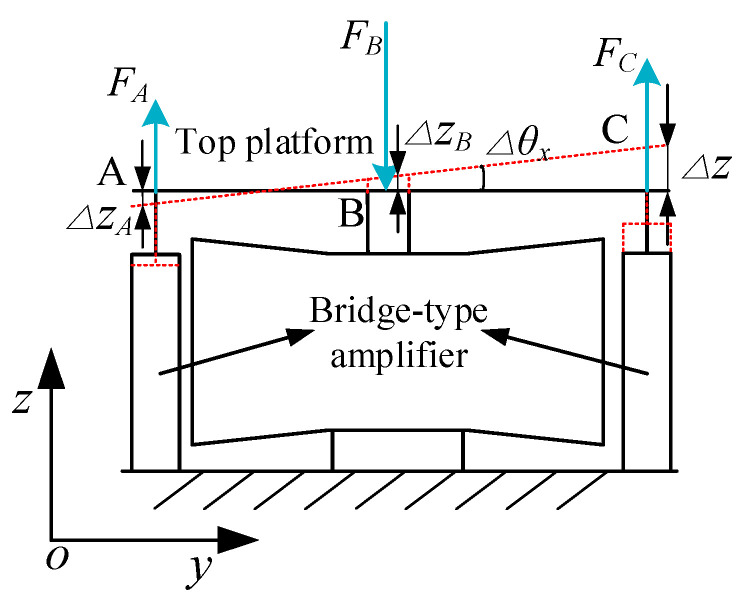
Schematic of rotation in the X-direction.

Due to the symmetrical structure of the platform, the rotation of the platform about the Y-direction is the same as the X-direction. As shown in Figure 7, when the right bridge-type amplifier is driven by the PZA, the platform has a rotation angle Δ*θ_x_* around the *X*-axis. At this point, the left bridge-type amplifier will be compressed, and the middle two will be stretched. According to the balance of forces, the following relation is obtained:

(20) $$F_{A}+F_{C}=F_{B}.$$

The equivalent stiffness of the bridge amplifier is known as *K_e_*, and the parallel stiffness of the two bridge amplifiers at the middle point B is 2*K_e_*. The following relations are obtained by Hooke’s law:

(21) $$F_{A}=K_{e}\Delta z_{A};\;F_{B}=2K_{e}\Delta z_{B};\;F_{C}=K_{e}\Delta z.$$

Since the top platform is more rigid than the flexible hinge, it can be regarded as a rigid body, and its geometric condition can be obtained as:

(22) $$\{\begin{array}{l} \frac{\Delta z_{A}}{\tan\Delta \theta_{x}}+\frac{\Delta z}{\tan\Delta \theta_{x}}=L_{4}\\ \frac{\Delta z_{A}}{\tan\Delta \theta_{x}}+\frac{\Delta z_{B}}{\tan\Delta \theta_{x}}=\frac{1}{2}L_{4} \end{array}.$$

It can be computed by Equations (20)–(22) that:

(23) $$\Delta z_{A}=0;\;\Delta z_{B}=\frac{1}{2}L_{4}\Delta \theta_{x};\;\Delta z=L_{4}\Delta \theta_{x}.$$

The system kinetic energy mainly includes the generation of the movements of the bridge-type amplifiers and the rotation of the top platform, which can be written as:

(24) $$T_{\theta x}=\frac{1}{2}M_{e}{\left(L_{4}\Delta {\dot{\theta }}_{x}\right)}^{2}+M_{e}{\left(\frac{L_{4}}{2}\Delta {\dot{\theta }}_{x}\right)}^{2}+\frac{1}{2}J_{T}\Delta {\dot{\theta }}_{x}{}^{2}.$$

The potential energy is mainly stored in the bridge-type amplifiers and can be modeled as:

(25) $$V_{\theta x}=\frac{1}{2}K_{e}{\left(L_{4}\Delta \theta_{x}\right)}^{2}+2\times \frac{1}{2}K_{e}{\left(\frac{L_{4}}{2}\Delta \theta_{x}\right)}^{2}.$$

**Figure 8:**
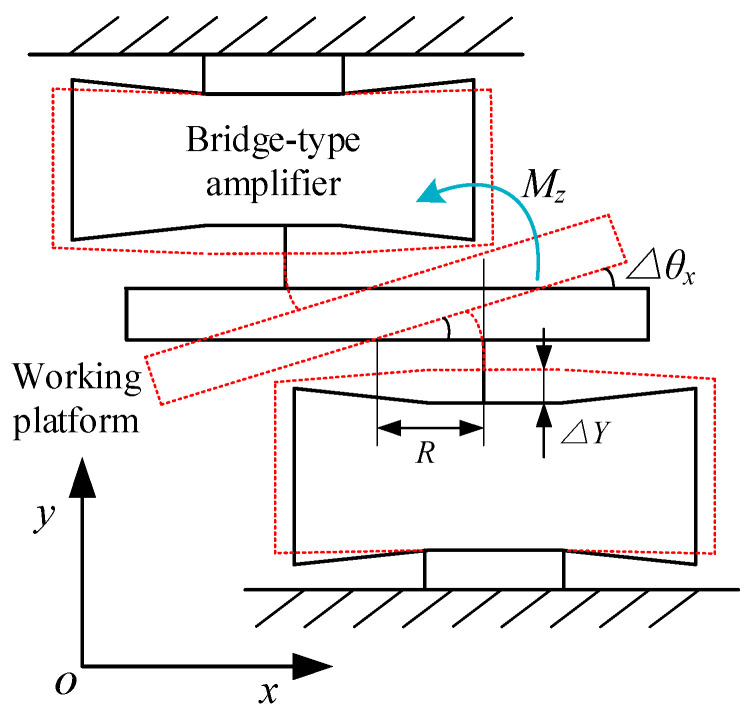
Schematic of rotation in the Z direction.

The rotation of the platform about the *Z*-axis is controlled by two bridge-type amplifiers on the top platform. As shown in Figure 8, when the output displacement of both bridge-type amplifiers of the top platform is Δ*Y*, the platform has a rotation angle Δ*θ_z_* around the *Z*-axis. According to the geometric relationship, the output displacement Δ*Y* of the bridge amplifier can be deduced as:

(26) $$\Delta Y=R\tan\Delta \theta_{z}\approx R\Delta \theta_{z}.$$

At this time, the system kinetic energy includes the moving kinetic energy of the bridge amplifier and the rotational kinetic energy of the working platform, which can be expressed as:

(27) $$T_{\theta z}=2\times \frac{1}{2}M_{e}{\left(R\Delta {\dot{\theta }}_{z}\right)}^{2}+\frac{1}{2}J_{5}\Delta {\dot{\theta }}_{z}{}^{2}.$$

Simultaneously, the system potential energy includes the elastic potential energy of the bridge-type amplifier and the bending deformation energy of the top platform flexible hinge *l*_5_, as follows:

(28) $$V_{\theta z}=2\times \frac{1}{2}K_{e}{\left(R\Delta \theta_{z}\right)}^{2}+2\times \frac{1}{2}k_{\theta 5}\Delta \theta_{z}{}^{2}$$

where *J*_5_ is the moment of inertia of the mass *m*_5_, and *k_θ_*_5_ is the bending stiffness of the top platform flexible hinge *l*_5_, which can be expressed as:

(29) $$k_{\theta 5}=\frac{Edt^{3}}{12l_{5}}.$$

Bringing the above kinetic energy and potential energy expressions into Equation (1), the dynamics model of the platform can be deduced as:

(30) $$\begin{array}{c} \left[\begin{array}{cccccc} M_{x}^{e} & 0 & 0 & 0 & 0 & 0\\ 0 & M_{y}^{e} & 0 & 0 & 0 & 0\\ 0 & 0 & M_{z}^{e} & 0 & 0 & 0\\ 0 & 0 & 0 & J_{\theta x}^{e} & 0 & 0\\ 0 & 0 & 0 & 0 & J_{\theta y}^{e} & 0\\ 0 & 0 & 0 & 0 & 0 & J_{\theta z}^{e} \end{array}\right]\left[\begin{array}{c} \ddot{x}\\ \ddot{y}\\ \ddot{z}\\ {\ddot{\theta }}_{x}\\ {\ddot{\theta }}_{y}\\ {\ddot{\theta }}_{z} \end{array}\right]+\left[\begin{array}{cccccc} C_{1} & 0 & 0 & 0 & 0 & 0\\ 0 & C_{2} & 0 & 0 & 0 & 0\\ 0 & 0 & C_{3} & 0 & 0 & 0\\ 0 & 0 & 0 & C_{4} & 0 & 0\\ 0 & 0 & 0 & 0 & C_{5} & 0\\ 0 & 0 & 0 & 0 & 0 & C_{6} \end{array}\right]\left[\begin{array}{c} \dot{x}\\ \dot{y}\\ \dot{z}\\ {\dot{\theta }}_{x}\\ {\dot{\theta }}_{y}\\ {\dot{\theta }}_{z} \end{array}\right]\\ +\left[\begin{array}{cccccc} K_{x} & 0 & 0 & 0 & 0 & 0\\ 0 & K_{y} & 0 & 0 & 0 & 0\\ 0 & 0 & K_{z} & 0 & 0 & 0\\ 0 & 0 & 0 & K_{\theta x} & 0 & 0\\ 0 & 0 & 0 & 0 & K_{\theta y} & 0\\ 0 & 0 & 0 & 0 & 0 & K_{\theta z} \end{array}\right]\left[\begin{array}{c} x\\ y\\ z\\ \theta_{x}\\ \theta_{y}\\ \theta_{z} \end{array}\right]=\left[\begin{array}{c} F_{x}\\ F_{y}\\ F_{z}\\ M_{x}\\ M_{y}\\ M_{z} \end{array}\right] \end{array}$$

Among them, the mass matrix unit is solved as:

(31) $$\{\begin{array}{c} M_{x}^{e}=M_{M}+M_{T}+2M_{e}+4M_{B}+\frac{8J_{4}}{{\left(2l_{3}+L_{3}\right)}^{2}}\\ M_{z}^{e}=M_{T}+4M_{e}\\ J_{\theta x}^{e}=J_{\theta y}^{e}=\frac{3L_{4}{}^{2}M_{e}}{2}+J_{T}\\ J_{\theta Z}^{e}=2R^{2}M_{e}+J_{5} \end{array}$$

The stiffness matrix unit is solved as:

(32) $$\{\begin{array}{c} K_{x}=K_{y}=2K_{e}+k_{3}+2k_{g}\\ K_{z}=4K_{e}\\ K_{\theta x}=K_{\theta y}=\frac{3}{2}K_{e}L_{4}{}^{2}\\ K_{\theta z}=2K_{e}R^{2}+2k_{\theta 5} \end{array}$$

The structural damping of the model is taken as the ratio damping. Through the modal test (as shown in Section 4.1), the modal damping ratio *ξ_i_* can be identified, and the damping matrix *[C]* can be obtained as:

(33) $$C_{i}=2\xi_{i}\sqrt{M_{ei}K_{i}}.$$

## 3. FEA Verification

### 3.1. Natural Frequency Verification of Bridge-type Amplifier

Because FEA has high precision, the result of its analysis can be considered to be an actual value. Therefore, the calculation result can be used as a reference standard. The parameters of the model are all listed in Table 1. The alloy spring steel (65Si_2_Mn) was used, the density was 7850 kg·m^−3^, the elastic modulus was E = 210 GPa, and the Poisson’s ratio was 0.28.

The table’s parameters are brought into the theoretical natural frequency of the bridge-type amplifier to solve using the calculation software MATLAB-2016a (MathWorks, Inc., Natick, MA, USA). From the results, the theoretical natural frequency of the Y-direction can be obtained as *f_n_* = 266.95 Hz. In addition, using the FEA software ANSYS18.2 (ANSYS, Inc., Canonsburg, PA, USA), the adaptive meshing method was used to establish a model that satisfied the convergence and accuracy requirements. The first six modes were obtained by modal simulation analysis of the bridge mechanism. 

**Figure 9:**
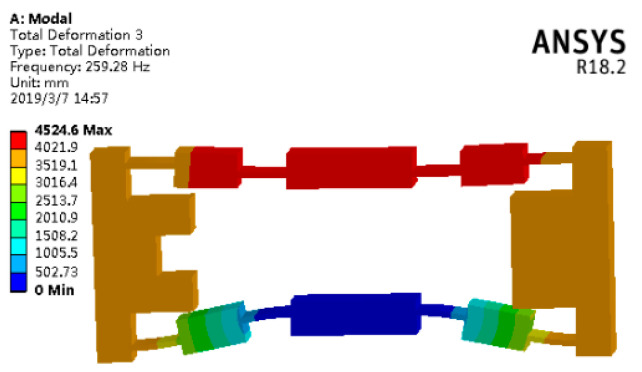
Third-order modal of vibration of the bridge-type amplifier.

Since only the translation along the Y direction is considered in the generalized coordinates of the analytical model, according to the vibration mode analysis, that was the third-order mode of the bridge mechanism. The third-order mode shape of the bridge-type amplifier is shown in Figure 9. It is easy to find that the FEA value of natural frequency was 259.28 Hz. Compared with the theoretical value of the natural frequency, 266.95 Hz, the error was only 2.96%, which fully proves the accuracy of the analytical model in this paper.

### 3.2. Natural Frequency Verification of Platform 

**Figure 10:**
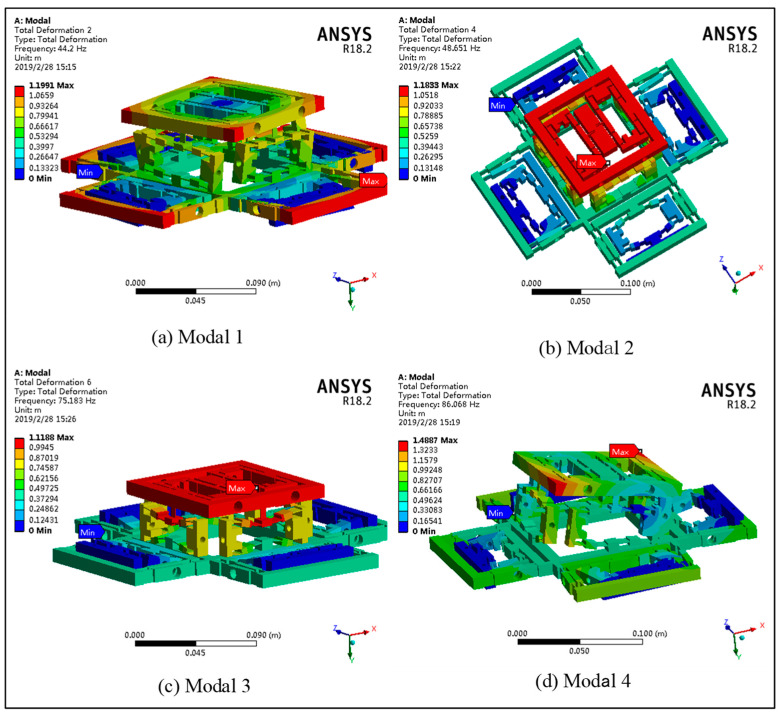
Modals of the finite element analysis (FEA). (**a**) The modal 1 of the platform; (**b**) the modal 2 of the platform; (**c**) the modal 3 of the platform; (**d**) the modal 4 of the platform.

**Figure 11:**
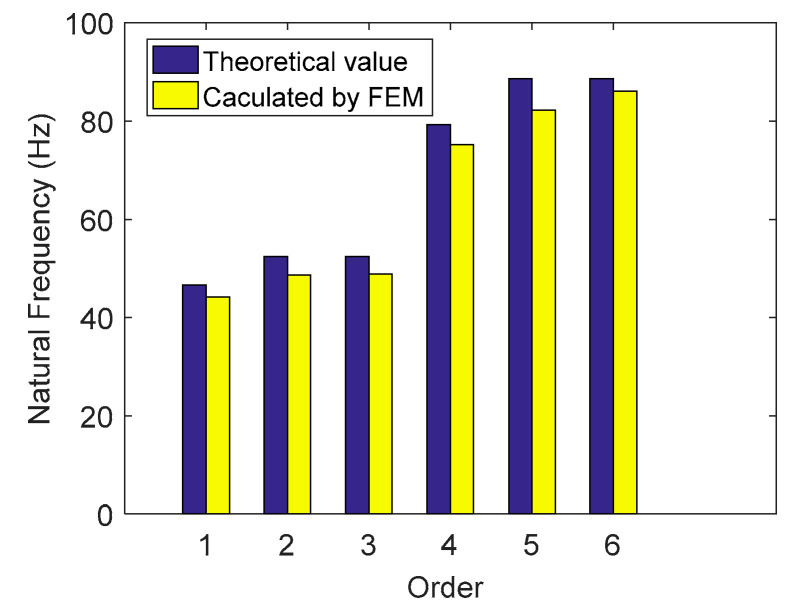
Comparison of the results between the theoretical and FEA.

The residual structural parameters of the platform are shown in Table 2. Alloy spring steel (65Si2Mn) was used as the material, the density was 7850 kg m^−3^, and the modulus of elasticity was 210 GPa. The Poisson’s ratio was 0.28. The natural frequency of the platform was obtained by solving the theoretical model. To verify the correctness of the theoretical model, the high precision simulation value of the natural frequency was obtained using the FEA software ANSYS18.2. The first four modes of FEA are shown in Figure 10. In addition, the comparison between theoretical value and FEA value is shown in Figure 11.

As seen in Figure 11, a certain error exists between the theoretical value and the FEA value; however, it is not large. The theoretical and FEA results of each order’s natural frequency are compared, and the error analysis is shown in Table 3. The maximum error, 7.81%, exists in modal 5, in which vibration mode is the X-rotation. The larger error may be that the coupling effect of the other direction, which is ignored, is slightly affected in the calculation of its stiffness and kinetic energy.

## 4. Experimental Verification

### 4.1. Modal Experiment

**Figure 12:**
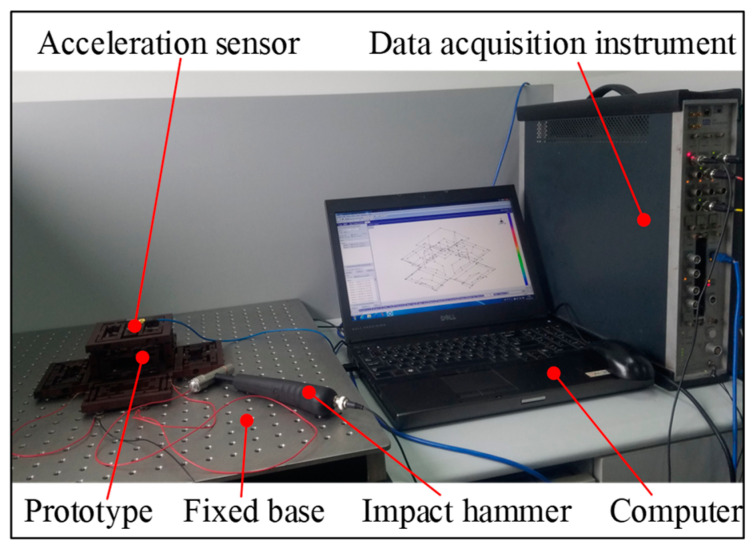
Schematic diagram of the modal test.

**Figure 13:**
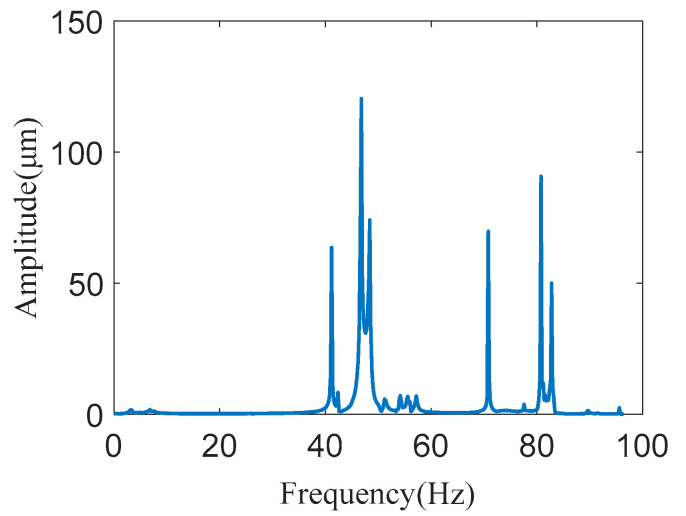
The spectrogram of the platform.

To further verify the natural frequency and identify the modal damping ratio, a modal experiment was performed. The experimental setup is shown in Figure 12. This includes the LMS Test Lab 9A test system (LMS International, Inc., Leuven, Belgium), LMS SCADAS-III (LMS International, Inc., Leuven, Belgium) data acquisition instrument, B&K 4525-B three-way acceleration sensor, and B&K 8206–002−54138 type excitation hammer (LMS International, Inc., Leuven, Belgium). When the test piece is struck by a hammer, the exciting force signal can be measured by a force sensor mounted inside it. The response signal of the platform was measured using a three-way acceleration sensor. Then, the sensor’s data were connected to the data acquisition, and the modal analysis was performed through the data acquisition and analysis system. The experimental results are shown in Figure 13. The peak of magnitude occurred at the natural frequency.

According to the platform’s modal experiment results, the platform’s modal parameters were analyzed and compared with the theoretical value, as shown in Table 4.

It can be seen from Table 4 that there was a specific error between the theoretical value and the experimental value of the first six natural frequencies of the platform, and the error was larger than the error between the FEA and the theoretical value. The maximum error was 8.64% at the fourth order natural frequency. The main reasons include the following: (1) errors in the manufacturing process, (2) the environmental disturbances during the experiment, (3) piezoelectric ceramics were not considered in the theoretical model, and (4) experimental operation error. In addition, the modal damping ratio of the platform was identified using a modal test, which can be used to solve the dynamic response of the model.

### 4.2. Step Response Experiment

To verify the rationality and correctness of the theoretical model, dynamic response experiments of the system were also performed. The step response test is generally used to reflect critical parameters, such as the dynamic response speed and system stability of the flexible positioning platform. The settling time *t_s_*, the step displacement *S_p_* and the maximum overshoot *M_p_* of the platform system were determined using the input step signal.

**Figure 14:**
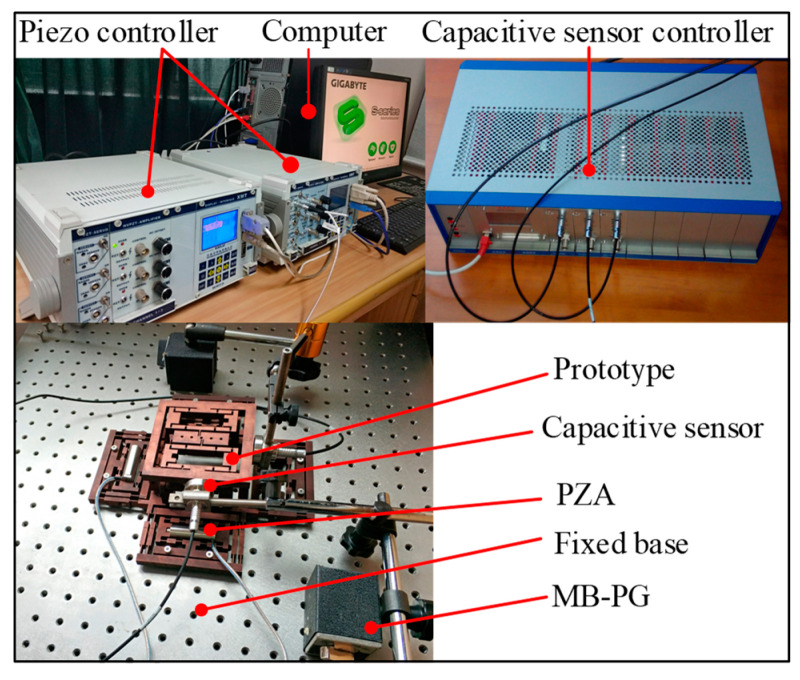
Schematic diagram of the experimental setup. (PZA: piezoelectric actuator; MB-PG: Magnetic metric base)

As shown in Figure 14, the devices, the piezoelectric controller of type E01, the PZA of model PSt−40VS15 (from COREMORROW, Harbin, China), and the capacitive displacement sensor of model CS5, from MICRO-EPSILION, Bavaria, Germany, measuring a range of 5 mm and a resolution of 100 nm, were used in this study. We inserted the PZA into the bridge-type amplifier and applied a preload force on both ends of the PZA through two screws to ensure a stable connection between the two. The fixing holes of the compliant platform were fixed to a fixed base mounted on the optical table to reduce ground vibration.

**Figure 15:**
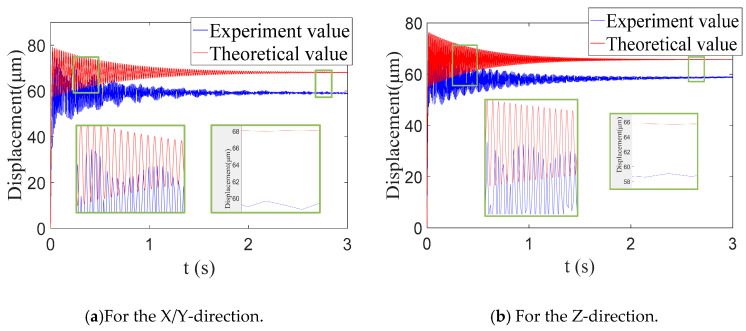
Step response for the system identification.

A 10 μm step signal was input to the PZA in the X-direction and the Z-direction. The symmetrical structure was such that the case in the Y direction was the same as that in the X direction; thus, there was no need to do it in the Y direction. Then, the displacement sensor was used to detect the output displacement of the compliant platform to obtain a step response. The step response measured by the test was compared with the theoretical value, as shown in Figure 15. The analytical dynamic model of the input step response was a second-order ordinary differential equation, which was solved by MATLAB using the fourth-order Runge–Kutta method. The allowable error band was taken to be ±2%, depending on the accuracy requirements. The step output displacement *S_p_* and settling time *t_s_* of the theory and experiment are all listed in Table 5.

Through analysis, the main reasons for the above errors were as follows: (1) There were installation errors during the installation of the whole test device, including the installation angle offset and the gap between the screw and the screw hole. (2) Since the compliant platform was monolithically fabricated by wire cut electrical discharge machining (WEDM) technology, there were corner radii and other manufacturing errors. (3) There were measurements affected by external environmental interference during the measurement process.

According to the theory of control engineering, when the allowable error is ±2%, the settling time of the system can be expressed as:

(34) $$t_{s}\approx \frac{4}{\xi \omega_{n}}=\frac{2}{\pi \xi f_{n}}$$

where $\omega_{n}$ is the natural angular frequency.

**Figure 16:**
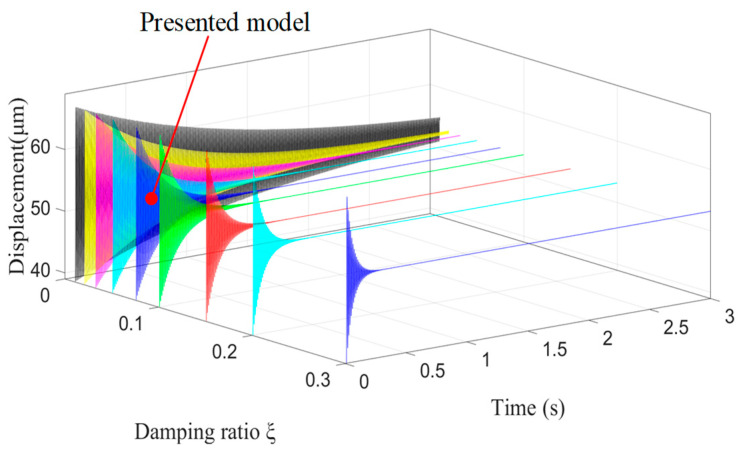
The vibration image of the Z-direction varying with the damping ratio *ξ.*

The above results reflect that the value of *t_p_* was too large. One of the reasons was the small natural frequency of the device. There was a positive correlation between the natural frequency and stiffness, and the frequency increased when the stiffness increased. Therefore, the response speed can be increased by maximizing its stiffness when designing a compliant platform. Another reason was the small damping of the structure and material of the device. Therefore, the response speed can also be increased by increasing the system damping. The vibration image of the Z-direction varying with the damping ratio is shown in Figure 16. The system settling time varied with the damping ratio, which is consistent with Equation (17) and proves the correctness of the theory.

## 5. Conclusions

This paper introduced the structure and principle of a 6-DOF compliant platform driven by a PZA based on a bridge-type amplifier. The dynamic model of the bridge-type amplifier was established using the Lagrange equation. FEA was used to verify the natural frequency of the bridge-type amplifier, and the error with the theoretical value was only 2.96%, which fully proves its correctness.

Based on the dynamics model of the bridge-type amplifier, the dynamic analysis model of each DOF of the compliant platform was established using the Lagrange equation under the condition of damping. FEA was used to analyze the modality of the compliant platform to obtain the natural frequency in each direction, and the maximum error was 7.81% compared with the theoretical value. 

Through the modal experiment, the damping ratio and natural frequency of the compliant positioning platform were identified. In addition, the dynamic response experiments of the system were conducted to further verify the rationality and correctness of the theoretical model. The maximum error of the settling time and the step output displacement were 13.34% and 17.55% compared with the theoretical results calculated by the fourth-order Runge–Kutta method. The analysis showed that the main cause of dynamic performance was system stiffness and damping; therefore, adequate stiffness and damping should be ensured when designing a compliant platform.

In the process of the research, we found that the amplification ratio of the whole compliant platform was much lower than that of a single bridge-type amplifier. One of the main reasons was that more elastic potential energy was stored in the guiding mechanism. In addition, the coupling displacement in other directions also dramatically weakened the output displacement. Therefore, more attention should be paid to this problem in follow-up studies.

## Figures and Tables

**Table 1:** The parameter values of the bridge-type amplifier model.

Parameters	L/mm	l/mm	v/mm	t/mm	d/mm	E/GPa
Value	9	7	1.2	0.8	10	210

**Table 2:** The platform’s structural parameters.

Parameters	L_1_	L_2_	L_3_	l_3_	l_5_	B	R
Value/mm	18	14	28	10	9	16	4

**Table 3:** Analysis results of the theory and FEA.

Order	Natural frequency *f_n_*/Hz			Mode of vibration
	Analytical	FEA	Error/%	
1	46.637	44.2	5.51	Rotation along Z axis
2	52.421	48.651	7.75	Translation along X axis
3	52.420	48.828	7.36	Translation along Y axis
4	79.246	75.183	5.40	Translation along Z axis
5	88.598	82.178	7.81	Rotation along X axis
6	88.598	86.068	2.94	Rotation along Y axis

**Table 4:** Analysis results of the theory and experiment.

Order	Natural frequency *f_n_*/Hz			Damping ratio *ξ/%*
	Theory	Experiment	Error/%	
1	46.637	41.2	11.66	0.28
2	52.421	46.8	10.72	0.73
3	52.420	48.4	7.671	0.86
4	79.246	70.8	10.66	0.16
5	88.598	80.2	9.479	0.79
6	88.598	82.1	7.334	0.34

**Table 5:** Comparison of the values between the theoretical and experimental.

Directions	Step output displacement *S_p_*/μm			Settling time *t_s_*/s		
	Theory	Experiment	Error	Theory	Experiment	Error
X/Y	68.01	58.94	13.34%	1.43	1.62	13.29%
Z	65.72	58.75	10.61%	1.31	1.54	17.55%
